# Glycosuria in primary glomerulopathies: prevalence and prognostic significance

**DOI:** 10.1590/2175-8239-JBN-2021-0115

**Published:** 2021-08-18

**Authors:** Carolina Ormonde, Ivo Laranjinha, Célia Gil, Margarida Gonçalves, August a Gaspar

**Affiliations:** 1 Hospital do Divino Espírito Santo, Avenida D.Manuel I, 9500-370, Ponta Delgada, Portugal.; 2 Hospital de Santa Cruz, Centro Hospitalar Lisboa Ocidental, Avenida Prof. Dr. Reinaldo dos Santos, 2790-134, Carnaxide, Portugal.

**Keywords:** Glycosuria, Glomerulonephritis, Prognosis, Albuminuria, Kidney Tubules, Proximal, Renal Insufficiency, Chronic, Glicosúria, Glomerulonefrite, Prognóstico, Albuminúria, Túbulos renais proximais, Insuficiência Renal Crônica

## Abstract

**Introduction:**

Tubular damage is common in glomerular diseases (GD). Glycosuria is a marker of tubular dysfunction and may be used to detect tubular lesion and CKD progression. The aim of this study was to evaluate the prevalence and prognostic value of glycosuria at the time of diagnosis in primary glomerulopathies (PG).

**Methods:**

We conducted a 24-month retrospective study in patients diagnosed with PG in our center between 2009 and 2020. We excluded diabetic patients, use of SGLT2 inhibitors, transplant patients, and secondary GD. Patients were divided in two groups according to their glycosuria status at diagnosis.

**Results:**

We studied 115 patients. Global prevalence of glycosuria was 10% (n=11) and membranous nephropathy (MN) had the highest prevalence (n=5, 17.9%). We found that patients with glycosuria had higher serum creatinine (2.4 vs. 1.2 mg/dL, p=0.030), higher albuminuria (4.8 vs. 1.9 g/g, p=0.004), and lower serum albumin (2.3 vs. 3.2 g/dL, p=0.021). We did not find association with histological prognostic factors. At the end of follow-up, patients with glycosuria had higher prevalence of the composite outcome of stage 5D CKD or 50% increase in basal SCr (45.5% vs. 17.3%, p=0.037). In patients with MN, results were similar but we were able to find an association of glycosuria with more severe interstitial fibrosis and tubular atrophy (25.0 vs. 0.0 %, p=0.032).

**Conclusion:**

Ten percent of our patients with PG have glycosuria. Glycosuria at the time of diagnosis was associated with more severe clinical presentation and worst renal outcome. The association with higher albuminuria suggests that tubular function has an impact on the severity and outcomes of PG.

## Resumo

**Introdução:**

Danos tubulares são comuns em doenças glomerulares (DG). Glicosúria é um marcador de disfunção tubular e pode detectar lesão tubular e progressão da DRC. O objetivo deste estudo foi avaliar a prevalência e o valor prognóstico da glicosúria no diagnóstico em glomerulopatias primárias (GP).

**Métodos:**

Realizamos estudo retrospectivo de 24 meses em pacientes diagnosticados com GP em nosso centro entre 2009-2020. Excluímos pacientes diabéticos, uso de inibidores de SGLT2, pacientes transplantados e DG secundárias. Os pacientes dividiram-se em dois grupos de acordo com seu estado de glicosúria no diagnóstico.

**Resultados:**

Estudamos 115 pacientes. A prevalência global de glicosúria foi de 10% (n=11) e a nefropatia membranosa (NM) teve maior prevalência (n=5, 17,9%). Constatamos que pacientes com glicosúria apresentavam creatinina sérica mais elevada (2,4 vs. 1,2 mg/dL, p=0,030), albuminúria mais alta (4,8 vs. 1,9 g/g, p=0,004), e albumina sérica mais baixa (2,3 vs. 3,2 g/dL, p=0,021). Não encontramos associação com fatores prognósticos histológicos. Ao final do acompanhamento, pacientes com glicosúria tiveram maior prevalência do desfecho composto de DRC estágio 5D ou aumento de 50% na CrS basal (45,5% vs. 17,3%, p=0,037). Em pacientes com NM, os resultados foram semelhantes, mas encontramos uma associação de glicosúria com fibrose intersticial mais grave e atrofia tubular (25,0 vs. 0,0 %, p=0,032).

**Conclusão:**

10% de nossos pacientes com GP têm glicosúria. A glicosúria no diagnóstico foi associada a uma apresentação clínica mais grave e pior desfecho renal. A associação com albuminúria mais elevada sugere que a função tubular tem um impacto na gravidade e nos desfechos da GP.

## Introduction

Glucose is freely filtered in the glomerulus and is almost completely reabsorbed in the proximal tubules by the sodium-glucose cotransporters (SGLT)^1^. Glycosuria, in euglycemic non-diabetic patients, is a known marker of proximal tubular disfunction^2^. It may be present with other Fanconi Syndrome manifestations as aminoaciduria, hyperuricosuria, hyperphosphaturia, and tubular acidosis^2^^,^ 3.

Glycosuria might be a valuable marker of prognosis in renal diseases. It is an extremely easy and inexpensive test. Besides, it seems to correlate with a more severe histological disease^2^^,^ 4. Glycosuria is reported to be increasingly more frequent as chronic kidney disease (CKD) progresses^1^. The vicious cycle mechanisms of CKD progression, with tubular inflammation and expansion of interstitial damage, are responsible for the appearance of tubular dysfunction markers in urinalysis, particularly glycosuria^2^^,^ 5. 

There are no studies comparing prevalence of glycosuria in various CKD etiologies. However, some studies have showed that glycosuria might be caused not only by CKD progression mechanisms but also by additional damage caused by filtered proteins in glomerular diseases (GD)^1^. Furthermore, it seems that the amount of proteinuria correlates with the risk for CKD progression^6^^,^ 7. Some studies have reported that glycosuria is not rare in GD and might be a marker of tubular dysfunction and worst prognosis^4^^,^ 8 ^,^ 9. 

With this in mind, we hypothesized that glycosuria might be an easy, inexpensive, and useful marker in primary glomerulopathies (PG) to detect a worse prognosis at the time of diagnosis. Very few and small studies have assessed the prevalence and relevance of glycosuria in PG in adults^2^. Therefore, our study aimed to evaluate the prevalence and prognostic value of glycosuria at the time of diagnosis in adults with PG, as well as to analyze its association with PG’s severity.

## Methods

### Study design

This was a single-center, 2-year retrospective cohort study of patients with biopsy-proven primary glomerulonephritis.

### Subjects and Methods

We selected all patients diagnosed with PG in our center between 2009 and 2020 - 115 were eligible for the study. We included patients with biopsy-proven minimal change disease (MCD), focal segmental glomerulosclerosis (FSGS), membranous nephropathy (MN), and IgA nephropathy (IgAN). The exclusion criteria were: patients with diabetes and glucose intolerance, use of SGLT2 inhibitors, under 18 years old, kidney transplant patients, and secondary glomerulopathies. Demographic and clinical data and laboratory and biopsy results were collected from patients’ medical records.

### Statistical analysis

Categorical variables are expressed as frequencies and continuous variables non-normally distributed as median values with interquartile range (IQR). Comparison between variables was performed using Wilcoxon test for non-normally distributed variables and X^2^ test for categorical variables. Survival curves were estimated by Kaplan-Meier analysis and compared by log-rank test. Logistic regression analysis was used for multivariable analysis. Data were analyzed using SPSS Statistics 25.0. Significant results were considered when p value was less than 0.05. 

## Results

We studied 115 patients with PG. Forty patients (34.8%) had IgAN, 28 (24.3%) MN, 27 (23.5%) FSGS, and 20 (17.4%) MCD. The baseline characteristics of patients enrolled in the study are described in Table 1.

**Table 1:** Baseline demographic, clinical, and analytical data

Characteristics	All (n=115)		Glycosuria group (n=11)		Without glycosuria group(n=104)		*p value*
Age - median (IQR), years	43.9	(33.6-58.5)	41.3	(30.4-58.2)	44.3	(33.8-59.0)	0.827
Male gender - n (%)	71	(61.7%)	9	(81.8%)	62	(59.6%)	0.150
Hypertension - n (%)	49	(42.6%)	6	(54.5%)	43	(41.3%)	0.626
Serum Creatinine - median (IQR), mg/dL	1.3	(1.0-2.0)	2.4	(1.2-4.0)	1.2	(0.9-1.8)	0.030
eGFR - median (IQR), mL/min/1.73m^2^	57.7	(37.9-91.0)	25.2	(12.9-69.1)	63.0	(39.8-91.9)	0.050
Albuminuria - median (IQR), g/g	2.3	(1.1-4.7)	4.8	(3.0-7.3)	1.9	(1.0-4.3)	0.004
Serum Albumin - median (IQR), g/dL	3.2	(2.0-4.0)	2.3	(1.8-2.7)	3.2	(2.0-4.1)	0.021
Microhematuria - n (%)	87	(75.7%)	10	(90.9%)	77	(74.0%)	0.215
Hemoglobin - median (IQR), g/dL	13.3	(12.5-14.6)	12.7	(11.0-13.4)	13.4	(12.6-14.7)	0.031
Serum Uric Acid - median (IQR), mg/dL	6.5	(5.1-8.3)	5.4	(4.3-6.5)	6.9	(5.2-8.3)	0.062
Serum Phosphate - median (IQR), mg/dL	4.1	(3.5-4.5)	4.4	(3.4-5.5)	4.0	(3.5-4.5)	0.194
%GS - median (IQR), %	10.0	(0.0-33.3)	9.1	(0.0-60.0)	10.6	(0.0-32.8)	0.546
IFTA - median (IQR), %	10.0	(0.0-30.0)	10.0	(0.0-40.0)	12.5	(0.0-30.0)	0.916

Global prevalence of glycosuria was 10% (n=11). Patients with MN had higher prevalence of glycosuria (n=5, 17.9%), followed by FSGS (n=3, 11.1%), IgAN (n=2, 5.0%), and MCD (n=1, 5.0%). These prevalence values were not statistically different (p=0.290). Also, demographic data were not different between patients with or without glycosuria (Table 1). 

### Baseline Analysis

We found that patients with glycosuria, compared with patients without, had higher serum creatinine (SCr) (2.4 vs. 1.2 mg/dL, p=0.030) and lower estimated glomerular filtration rate (eGFR) (25.2 vs. 63.0 mL/min/1.73 m^2^, p=0.050), higher albuminuria (Ualb) (4.8 vs. 1.9 g/g, p=0.004) and lower serum albumin (Salb) (2.3 vs. 3.2 g/dL, p=0.021). We also found lower hemoglobin levels in patients with glycosuria (12.7 vs. 13.4 g/L, p=0.031).

In a multivariate analysis, glycosuria was positively associated with MN diagnosis (OR 10.9, 95% CI 1.490-79.020, p=0.019), SCr (OR 2.6, 95% CI 1.074-6.317, p=0.034), and Ualb (OR 1.0, 95% CI 1.000-1.001, p=0.016), but not with hematuria, proportion of sclerotic glomerulus (%SG), and interstitial fibrosis and tubular atrophy (IFTA).

Nevertheless, we did not find statistically significant differences between patients with and without glycosuria in histological markers of chronicity, such as proportion of %SG or IFTA (Table 1).

### Follow-up analysis

**Figure 1:**
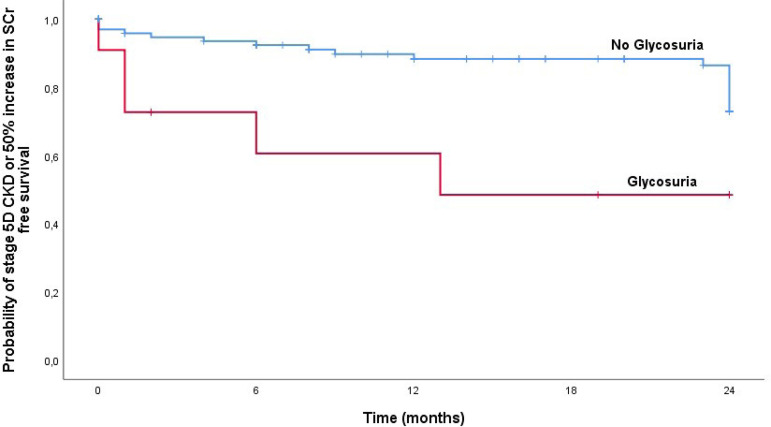
Renal survival (5D CKD stage or 50% increase in SCr) by Kaplan-Meier analysis for all primary glomerulopathies.

The follow-up period analysis is described in Table 2. Median follow-up was 19 (6-24) months. Patients with glycosuria showed worst renal outcomes with higher prevalence of the composite outcome of stage 5D CKD or 50% increase in basal SCr (45.5% vs 17.3%, p=0.037). In a Kaplan-Meier analysis (Figure 1) we confirmed that patients without glycosuria had a longer period to reach stage 5D CKD or 50% increase in SCr (log-rank test, p=0.011). Cox-regression analysis confirmed these results in a model adjusted for age, hypertension, immunosuppression therapy, and Ualb at baseline (HR=4.461, 95% CI 1.319-15.088, p=0.016). We did not find differences between the two groups in 1-year and 2-year SCr and eGFR decline rate or Ualb. 

**Table 2:** Twenty-four-month follow-up analysis

Characteristics	Glycosuria group		Without glycosuria group		p value
Immunosuppressive therapy - n (%)	7	(63.6%)	59	(56.7%)	0.766
1-y SCr - median (IQR), mg/dL	1.2	(1.0-1.7)	1.2	(0.8-1.6)	0.467
2-y SCr - median (IQR), mg/dL	1.5	(1.2-1.1)	1.2	(0.8-1.6)	0.243
eGFR decline rate - median (IQR), mL/min/1.73m^2^/year	-0.6	(-14.1-4.2)	0.6	(-2.0-5.0)	0.648
1-y Ualb - median (IQR), g/g	0.6	(0.3-1.8)	0.4	(0.08-1.4)	0.693
2-y Ualb - median (IQR), g/g	2.0	(0.3-3.7)	0.3	(0.08-0.9)	0.156
2-y stage 5D CKD or 50% rise in SCr - n (%)	5	(45.5%)	18	(17.3%)	0.037

1-y - 1 year; 2-y - 2 years

Glycosuria evolution through follow-up was variable - some patients resolved and some maintained. We could not find any association between glycosuria evolution and prognostic factors.

### Membranous nephropathy

Once MN is the PG with higher prevalence of glycosuria, we conducted a sub-analysis in this group of patients. The results are presented in Table 3. Similarly to the global analysis, MN patients with glycosuria had higher baseline SCr (2.4 vs. 1.0 mg/dL, p=0.021) and higher Ualb (7.1 vs. 3.5 g/g, p=0.033). They also presented lower eGFR at baseline (25.2 vs. 89.9 mL/min/1.73m^2^, p=0.021) and lower hemoglobin (12.2 vs. 13.5 g/L, p=0.015) than non-glycosuria MN patients. 

**Table 3:** Baseline characteristics of patients with membranous nephropathy

Characteristics	All		Glycosuria group		Without glycosuria group		p value
	(n=28)		(n=5)		(n=23)		
Age - median (IQR), years	51.5	(41.3-60.4)	52.8	(40.6-67.0)	50.2	(41.3-60.4)	0.928
Male gender - n (%)	20	(71.4%)	4	(80.0%)	16	(69.6%)	0.640
Hypertension - n (%)	12	(42.9%)	4	(80.0%)	8	(34.8%)	0.064
Serum Creatinine - median (IQR), mg/dL	1.1	(0.9-1.7)	2.4	(1.3-3.5)	1.0	(0.8-1.6)	0.021
eGFR - median (IQR), mL/min/1.73m^2^	72.1	(41.8-96.5)	25.2	(18.8-58.9)	89.9	(51.4-98.6)	0.021
Albuminuria - median (IQR), g/g	3.8	(2.8-6.7)	7.1	(4.5-15.4)	3.5	(2.4-5.0)	0.033
Serum Albumin - median (IQR), g/dL	2.4	(1.9-3.0)	2.6	(1.9-3.1)	2.4	(1.8-2.7)	0.950
Microhematuria - n (%)	18	(64.3%)	4	(80.0%)	14	(60.9%)	0.418
Hemoglobin - median (IQR), g/dL	13.3	(12.3-13.9)	12.2	(10.3-12.8)	13.5	(12.8-14.5)	0.015
Glomerulosclerosis - median (IQR), %	0.0	(0.0-17.3)	9.1	(0.0-59.8)	0.0	(0.0-9.7)	0.173
IFTA - median (IQR), %	0.3	(0.0-28.8)	25.0	(5.3-50.0)	0.0	(0.0-20.0)	0.032

In contrast to the global baseline values, we were able to show that MN patients with glycosuria had higher IFTA (25.0 (5.3-50.0) vs. 0.0 (0.0-20.0) %, p=0.032) when compared to non-glycosuria MN patients. 

At the end of the follow-up period there was no difference between groups in all outcomes (SCr, Ualb, eGFR decline and progression to 5D CKD or 50% rise in SCr). 

## Discussion

Glycosuria was found at presentation in 10% of patients with PG. Glycosuria was associated with a more severe clinical presentation of PG (with lower eGFR, higher levels of Ualb, and lower Salb). We also found that patients with glycosuria had poorer renal prognosis with higher risk of CKD progression than non-glycosuria group. The prevalence of glycosuria in MN patients (18%) was higher than in other PG. At the same direction, MN patients with glycosuria presented a more severe disease at diagnosis, including higher IFTA, than non-glycosuria MN patients.

Similar studies reported higher prevalence of glycosuria in PG (28%)^2^^,^ 4. Our lower prevalence may be associated with the fact that we only accounted as glycosuric patients those with glycosuria at the time of diagnosis, whereas other studies have accounted it when present over various periods of the disease. Other studies also reported MN as the PG with higher glycosuria prevalence (up to 50%)^4^^,^ 8.

Few investigations have studied the impact of glycosuria in PG. Woronik et al. (1998)^4^ studied 60 patients with PG retrospectively and Praga et al. (1991)^2^ investigated tubular disfunction in 36 nephrotic patients. Glycosuria was much more frequent than other manifestations of Fanconi Syndrome^2^. Similar to our findings, these studies reported higher SCr levels, higher Ualb, and lower Salb in the glycosuria group. Additionally, they confirmed higher levels of IFTA and worst renal outcomes in patients with glycosuria^2^^,^ 4. 

Some investigations suggest that tubulointerstitial lesions may correlate better with outcomes in PG than glomerular damage itself^10^^,^ 11. They hypothesize that this glomerular-tubular relationship, that might be mediated by albuminuria, could be bidirectional. Tubular damage may be both cause and consequence of higher albuminuria and worst renal outcomes in PG. Also, there are case reports showing that tubular function has a prominent impact on albuminuria levels - patients with typical tubular renal diseases present albuminuria even in the absence of glomerular lesions^3^^,^ 12.

There are several hypotheses about the mechanisms of tubular dysfunction in PG. The most widely accepted is that there is an association between the amount of filtered proteins and the degree of tubular damage^13^. After being filtered, proteins (including albumin) are reabsorbed in the proximal tubules by endocytosis, mainly in S1 segment^9^^,^ 10 ^,^ 12 ^,^ 14. Reubi et al. (1984)^8^ performed glucose titration experiments in 20 nephrotic patients and found that patients that present glycosuria show impaired kinetics of the proximal tubules^8^. In proteinuric diseases, such as GD, overload of filtered glomerular proteins overcomes tubular reabsorption capacity^1^^,^ 9 ^,^ 15. Nowadays it is recognized that the amount of filtered proteins is much higher than previously thought and that its reabsorption by the proximal tubule is essential to determine the excreted amount^14^^,^ 16 ^,^ 17. 

There is a complex crosstalk between glomerular and tubular cells^13^^,^ 18. The overload of proteins and the excessive endocytosis triggers tubular damage through the activation of several inflammatory molecules and pathways: complement activation molecules (mainly C3), chemokines (especially MCP-1), vasoactive agents (endothelin-1), and reactive oxygen species. Which filtered proteins (albumin or others) are responsible for these mechanisms is not known^9^^,^ 11 ^,^ 19. This inflammation leads to tubular and interstitial infiltration of inflammatory cells, particularly T-cells and macrophages. Additionally, other mechanisms such as tubular lumen obstruction by proteins and casts and production of other inflammatory molecules by damaged glomeruli may also contribute to this process^9^^,^ 11. This cascade induces continuous inflammation and activation of fibroblasts in all renal compartments, perpetuating a vicious circle^1^^,^ 18.

Thus, both glomerular and tubular function contribute to protein urinary excretion. Proximal tubular defects might affect GD pathophysiology and prognosis^13^. 

Another interesting fact presented in the literature is that glycosuria declines or resolves after remission of the nephrotic syndrome^4^^,^ 8. In fact, glycosuria (as a marker of tubular dysfunction) may be present at the beginning of the disease but it can be reversible if inflammatory lesions do not progress to fibrosis^13^^,^ 20. However, the persistence of glycosuria may be a marker of chronic fibrotic lesions in progressive CKD.

Our study had several limitations: it was a single-center study, which makes generalizability limited, a small retrospective cohort, with different types of histological GD included, which might confer heterogeneity to the sample, and our follow-up may be considered too short to evaluate renal outcomes in PG.

## Conclusion

In conclusion, glycosuria might be an easy, inexpensive, and useful marker in PGs to detect a more severe disease at diagnosis and prepare for a worst long-term prognosis. Prospective and longer studies are needed to confirm our results.
